# Joint Effects of *PON1* Polymorphisms and Vegetable Intake on Ischemic Stroke: A Family-Based Case Control Study

**DOI:** 10.3390/ijms18122652

**Published:** 2017-12-07

**Authors:** Juan Juan, Xia Jiang, Xun Tang, Yiqun Wu, Kexin Sun, Xiao Xiang, Yaohua Tian, Tao Wu, Qi Sun, Peter Kraft, Yonghua Hu

**Affiliations:** 1 Department of Epidemiology and Biostatistics, School of Public Health, Peking University, Beijing 100191, China; juanjuan@bjmu.edu.cn (J.J.); tangxun@bjmu.edu.cn (X.T.); qywu118@163.com (Y.W.); kexin_sun@bjmu.edu.cn (K.S.); doublex1990@live.cn (X.X.); 13036100424@163.com (Y.T.); twu@bjmu.edu.cn (T.W.); 2 Program in Genetic Epidemiology and Statistical Genetics, Harvard T.H. Chan School of Public Health, Harvard University, Boston, MA 02115, USA; xiajiang@hsph.harvard.edu (X.J.); pkraft@hsph.harvard.edu (P.K.); 3 Department of Nutrition, Harvard T.H. Chan School of Public Health, Harvard University, Boston, MA 02115, USA; qisun@hsph.harvard.edu; 4 Channing Division of Network Medicine, Department of Medicine, Brigham and Women’s Hospital and Harvard Medical School, Harvard University, Boston, MA 02115, USA; 5 Department of Epidemiology, Harvard T.H. Chan School of Public Health, Harvard University, Boston, MA 02115, USA

**Keywords:** gene-diet interaction, *PON1*, vegetable intake, ischemic stroke, ischemic stroke subtypes, family-based case-control study

## Abstract

Paraoxonase 1 gene (*PON1*) polymorphisms and dietary vegetable and fruit intake are both established determinants of ischemic stroke (IS). However, little is known about whether these factors jointly influence the risk of IS. We analyzed the main effects of *PON1*, as well as the interactions between *PON1* and dietary vegetable or fruit intake with the risk of total IS and its subtypes in a family-based case-control study conducted among 2158 Chinese participants (1007 IS cases and 1151 IS-free controls) from 918 families. Conditional logistic regression models, with each family as a stratum, were used to examine the association between rs662 and IS. Gene-diet interactions were tested by including a cross-product term of dietary vegetable or fruit intake by rs662_G allele count in the models. Each copy of the *PON1* rs662_G allele was associated with 28% higher risk of total IS (*p* = 0.008) and 32% higher risk of large artery atherosclerosis subtype (LAA) (*p* = 0.01). We observed an interaction between rs662 and vegetable intake for both total IS (*p* = 0.006) and LAA (*p* = 0.02) after adjustment for covariates. Individuals who carry the rs662_A allele may benefit to a greater extent from intake of vegetables and thus be more effectively protected from ischemic stroke, whereas carriers of the G allele may still remain at greater risk for ischemic stroke due to their genetic backgrounds even when they consume a high level of vegetables. More studies are needed to replicate our findings among other populations.

## 1. Introduction

Stroke is a major cause of death and disability worldwide [1,2,3]. According to the data from Global Burden of Diseases, Injuries, and Risk Factors Study 2010 (GBD 2010) [4], stroke ranks as the second leading cause of death after ischemic heart disease, and the third leading cause of Disability-Adjusted Life Years (DALYs) lost [5]. Ischemic stroke (IS), accounting for approximately 87% of total strokes [6], is a complex multifactorial disease with genetic components, environmental triggers, and gene-environment interactions involved in its etiology. Ischemic stroke can be classified into five subtypes according to the Trial of Org 10172 in Acute Stroke Treatment (TOAST) criteria [7] based on their etiological mechanisms: large artery atherosclerosis (LAA), small arterial occlusion (SAO), cardio embolism (CE), stroke of other determined causes (OC), and stroke of undetermined causes (UND).

Circulating paraoxonase-1, a potential clinically useful biomarker for IS diagnosis [8,9,10,11], has been reported to inhibit the progression of atherosclerosis and lower the risk of stroke by protecting lipoproteins against oxidative modification [12,13,14]. The lead coding polymorphism of paraoxonase 1 gene (*PON1*) (rs662), located on the long arm of chromosome 7 at q21.3, encodes a non-synonymous amino acid substitution (A to G allele) and is the major determinant of paraoxonase-1 catalytic efficiency and concentration [14,15,16,17]. The association between *PON1* rs662 and risk of IS has been well documented by previous meta-analyses [18,19]. For example, a meta-analysis including 7384 cases and 11,074 controls found that *PON1* rs662_G was positively associated with risk of IS: the summary odds ratio (OR) (95% confidence interval (95% CI) was 1.10 (1.04–1.17) [18]. Similarly, another meta-analysis conducted among Caucasian, Japanese, and Chinese populations reported a significantly increased risk of IS for rs662_G carriers (OR: 1.21, 95% CI: 1.08–1.35, *p* = 0.0009) [19].

In addition to *PON1* polymorphisms, dietary factors also play an important role in the development of IS. Intake of vegetables and fruit has been consistently observed to be associated with lower risk of IS [20,21]. A recent meta-analysis reported that the pooled relative risks (RRs) of stroke comparing the highest versus lowest intake levels were 0.82 (95% CI: 0.77–0.87) for fruit, and 0.87 (95% CI: 0.81–0.95) for vegetables [22]. Another meta-analysis of cohort studies showed that compared with individuals who had less than three servings of fruit and vegetables per day, the pooled RRs of stroke were 0.89 (95% CI: 0.83–0.97) for those who consumed 3–5 servings/day, and 0.74 (95% CI: 0.69–0.79) for those who had >5 servings/day [23].

Although the association of single nucleotide polymorphisms (SNPs) in *PON1* and dietary intake of vegetables and fruit with the risk of IS has been well-established, little is known about whether *PON1* confers risk for different IS subtypes and how *PON1* polymorphisms and dietary vegetable or fruit jointly influence the risk of IS, especially for the Chinese population. Evaluating these associations provides important implications for understanding the risk of different disease phenotypes (e.g., IS subtypes) in individuals with different genotypes. Differential associations between diet and IS risk by genotype may inform personalized recommendations for consumption of vegetable and fruit. Therefore, the aim of our current study is to explore the association between *PON1* genotypes and IS subtypes, as well as the interactions between *PON1* genotypes and dietary intake of vegetable or fruit on IS in the Chinese population using a family-based design.

## 2. Results

The characteristics of the participants according to IS status are presented in Table 1. Compared with IS-free controls, the cases were older (*p* < 0.001), had a higher proportion of males (*p* < 0.001), were more likely to be smokers (*p* = 0.006), had a higher prevalence of hypertension (*p* < 0.001), and were more susceptible to type 2 diabetes (*p* < 0.001). Furthermore, the IS cases consumed less fruits than controls (*p* = 0.004). However, Body mass index (BMI) and vegetable intake were comparable between the two groups.

Each copy of the *PON1* rs662_G allele was associated with 28% higher risk of total IS (OR: 1.28, 95% CI: 1.07–1.54, *p* = 0.008) and 32% higher risk of LAA (OR: 1.32, 95% CI: 1.06–1.64, *p* = 0.01). We observed an increased yet non-significant risk of *PON1* rs662_G with SAO (OR: 1.46, 95% CI: 0.96–2.22, *p* = 0.08). However, we did not find any significant associations for the remaining three subtypes (CE, OC, and UND) (OR: 0.80, 95% CI: 0.41–1.53, *p* = 0.50) (Table 2).

**Figure 1:**
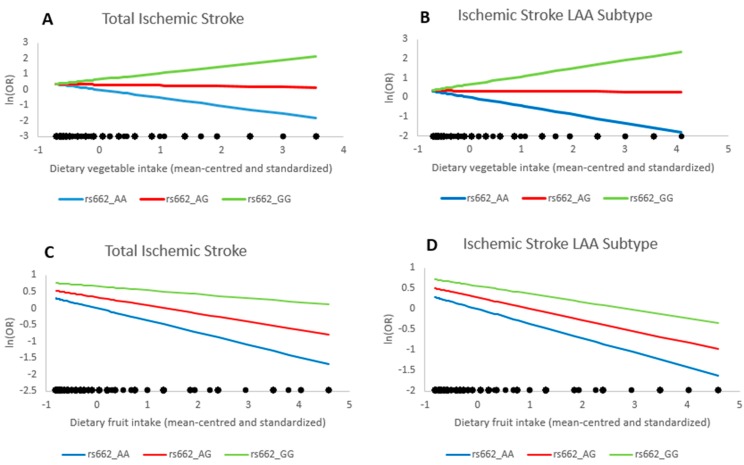
Interactions between *PON1* rs662_G allele numbers and standardized vegetable or fruit intake for total ischemic stroke and LAA. Interactions between *PON1* rs662_G allele count and vegetable intake for total ischemic stroke (**A**) and LAA (**B**) and interactions between *PON1* rs662_G allele count and fruit intake for total ischemic stroke (**C**) and LAA (**D**). Blue line represents those who are homozygous for the A allele (A/A), red line represents heterozygous (A/G), and green line represents homozygous for the G allele (G/G). The *Y*-axis shows the natural log transformed ORs. The *X*-axis shows the mean-centered and standardized intake levels of vegetable or fruit (participants with the highest and lowest 1% of vegetable intake levels were excluded to minimize the potential impact of outliers). LAA, large artery atherosclerosis; OR, odds ratio.

We found an interaction between *PON1* rs662 and vegetable intake for both total IS (*p* = 0.001) and LAA (*p* = 0.003) in our crude model (Table 3, Model 1). When adjusted for personal characteristics and lifestyle factors in the multivariate model (Model 2), the interactions remained statistically significant, although the magnitudes attenuated slightly (*p* = 0.006 for IS and *p* = 0.02 for LAA, respectively). After further adjustment for fruit intake, the interactions became non-significant with wider confidence intervals (Table 3, Model 3). Figure 1 shows the magnitude of *PON1*-vegetable and *PON1*-fruit interactions for both total IS and LAA. Among participants who carried rs662_AA genotype, each standard deviation increment in vegetable intake was associated with an average of 40% and 36% decreased risk of total IS and LAA, respectively. For participants with the rs662_GG genotype, each standard deviation increment in vegetable intake was associated with an average of 51% and 52% increased risk of total IS and LAA, respectively (Figure 1A,B). Although we did not find any statistically significant interactions between *PON1* rs662 and fruit intake for total IS and its subtypes (Table 3), the magnitude of negative association between consumption of fruit and risk of total ischemic stroke and LAA subtype increased with each copy of the rs662_A allele (Figure 1C,D).

## 3. Discussion

In our family-based case-control study among the Chinese population, we observed that the *PON1* rs662_G was related to a higher risk of total IS and LAA. Furthermore, this gene-disease association could be modulated by vegetable intake. In particular, individuals who carry the rs662_A allele might benefit to a greater extent from higher consumption of vegetables and thus be more effectively protected from ischemic stroke than carriers of G allele.

The association between *PON1* and ischemic stroke has been investigated in several previous studies [18,19,24,25,26,27]. For example, a meta-analysis including 7384 cases and 11,074 controls, showed that *PON1* rs662_G was positively associated with risk of IS with summary OR (95% CI) of 1.10 (1.04–1.17) [18]. Similarly, a systematic review and meta-analysis conducted among Caucasian, Japanese, and Chinese populations reported a significantly increased risk of IS for rs662_G carriers (OR: 1.21, 95% CI: 1.08–1.35, *p* = 0.0009) [19], which was largely concordant with our marginal association results. However, a few previous studies conducted in the Chinese population failed to identify significant associations between *PON1* rs662 and IS [28,29,30] probably due to limited statistical power (total sample size ranged from 306 to 1016), and lack of classification of stroke subtypes (a mixed sample of potentially heterogeneous disease subtypes). Our results, based on a large sample size of 2158 participants, confirmed this gene-disease association in a Chinese population with adequate statistical power. We were also able to explore the association across IS subtypes, which previous studies did not have the opportunity to. Our rationale for examining different IS subtypes was based on their distinct underlying etiological mechanisms [7]. For example, LAA is caused by plaque formation, stenosis or occlusion of a major brain artery or branch carotid arteries due to atherosclerosis; whereas SAO is due to the involvement of small perforating end-arteries within the brain; CE, different from the former two, is secondary to embolus arising in the heart and traveling to the brain [7]. We observed that the rs662_G allele was more strongly associated with a higher risk of LAA, a phenotype shares similar etiology with atherosclerosis [7]. This is biologically plausible due to the fact that the rs662_A-encoded glutamine-containing (Q) paraoxonase is more effective in exerting anti-oxidant and anti-atherogenic effects through hydrolyzing peroxides and lactones [8,31,32] than rs662_G-encoded arginine-containing (R) paraoxonase [17,33,34,35].The Q paraoxonase helps protect against the induction of monocyte-endothelial interactions in artery wall and therefore inhibits the development of atheroma, atherosclerosis, eventually decreases the risk of LAA [8,13,36]. However, downstream functional experiments should be performed to elucidate the biological mechanisms of the link observed between this polymorphism and IS.

An interesting finding of our study is that vegetable intake could modulate the association of *PON1* rs662 with both total IS and LAA. To the best of our knowledge, our study is the first investigation that evaluated and demonstrated such an interaction on ischemic stroke in the Chinese population. The literature on gene-diet interaction with regard to vegetable intake in the risk of ischemic stroke is sparse. One previous study conducted in the Atherosclerosis Risk in Communities (ARIC) found folate, a common component of vegetable, jointly influences the risk of IS with *PON1* rs662 among white participants [37], which was in line with and partly supported by our results. However, the ARIC study investigated only one of the many nutritional components of vegetables instead of total vegetables. Although the underlying mechanisms for the observed interaction remain unclear, our findings are biologically plausible. Vegetables, rich in polyphenols, flavonoid and other antioxidants (such as vitamins and minerals) [38,39,40], have been reported to be highly effective in modulating the activity and concentration of paraoxonase 1 [41,42,43] by reducing oxidative stress and affecting *PON1* gene expression. For example, dietary polyphenols could increase *PON1* activity through their antioxidant properties and modulate gene expression through aryl hydrocarbon receptor (AhR)-activating properties [44,45,46,47,48]. As *PON1* exerts its anti-atherogenic effects primarily by protecting against oxidative modification, intake of vegetables enhances this ability. Carriers of the rs662_A allele who consume higher levels of vegetables may therefore benefit from the joint effects of genotype and diet and have a reduced risk of ischemic stroke. However, for individuals with the GG genotype, increased intake of vegetables still could not outweigh their susceptibility to ischemic stroke, probably because of the pronounced oxidative stress and impaired anti-oxidative protection due to their strong genetic risk factors. Another possible explanation could be the rs662_G encoded arginine-containing (R) paraoxonase and rs662_A coded glutamine-containing (Q) paraoxonase have different structures and mechanisms in exerting anti-oxidant and anti-atherogenic effects [33], which leads to distinct rs662-vegetable interaction mechanisms for carriers of different genotypes. However, further functional investigations on the potential mechanism are warranted to explain the underlying reasons.

Our current study is a well-conducted, large family-based case-control study of ischemic stroke in the Chinese population. Our family-based design provides a useful complementary strategy to avoid false positive results in traditional case-control studies owing to its robustness to population stratification [49,50]. In addition, both the dietary and lifestyle questionnaires in our study were completed by trained interviewers in face-to-face surveys instead of self-administered questionnaires therefore improving the data quality. Several limitations should also be considered. First, our results might be potentially confounded by unmeasured or unknown factors, although we have carefully adjusted for demographic and lifestyle risk factors of cardiovascular disease, as well as dietary factors. Second, measurement errors in the assessment of dietary intake are inevitable, although our semi-quantitative food frequency questionnaires (sFFQs) have been validated and reproduced. Moreover, the random measurement errors in dietary assessment might lead to an attenuation of true association. Third, the total energy intake cannot be calculated in the current study. However, we additionally controlled for other dietary factors, such as red meat, egg, milk, and cereal intake in the sensitivity analysis, and found no change in the results.

## 4. Materials and Methods

### 4.1. Study Population

Our family-based case-control study was conducted in Fangshan, a rural district located in the southwest of Beijing, China. Details of the Fangshan Family-based Ischemic Stroke Study in China (FISSIC) have been described elsewhere [51]. Briefly, we recruited ischemic stroke patients as probands, who satisfied the following inclusion criteria: (1) confirmed ischemic stroke patients; (2) at least 18 years old at the time of enrollment; (3) had at least one full sibling alive who can participate in this study. Probands whose self-reported ischemic stroke could not be confirmed by medical records, computerized tomography (CT), or magnetic resonance imaging (MRI); who were diagnosed of iatrogenic ischemic stroke associated with surgical/interventional procedures, such as coronary artery bypass grafting, carotid endarterectomy, or heart valve surgery; or who were diagnosed of ischemic stroke associated with autoimmune condition or endocarditis were excluded from our study. All biological siblings of the probands, who were older than 18 years old, were recruited by proband-initiated contact method [52]. Step siblings and adopted siblings were not eligible in our study. A total of 2158 participants (1007 IS cases and 1151 IS-free controls) from 918 families were included in our current study.

This study has been approved by the Peking University Institutional Review Board (IRB00001052-13027), Beijing, China (22 July 2013). Written informed consents were signed by every subject before they participated in the study. The study was conducted in accordance with the Declaration of Helsinki.

### 4.2. Ascertainment of Ischemic Stroke Cases and IS-Free Controls

Self-reported cases of ischemic stroke were confirmed through medical records review, including medical history, physical examinations, laboratory tests and results of CT and/or MRI. Physicians, who were blinded to the exposure status, reviewed the medical records and confirmed the diagnosis of ischemic stroke according to the criteria of National Survey of Stroke which require evidence of a constellation of neurological deficits of sudden or rapid onset lasting for greater than 24 h or until death due to thrombotic or embolic occlusion of a cerebral artery [53]. The confirmed IS patients were further classified into five subtypes according to the TOAST criteria [7] (LAA, SAO, CE, OC, and UND). In our study, LAA (*n* = 709) and SAO (*n* = 185) accounted for 70.4% and 18.4% of total IS cases, respectively. The remaining three subtypes (CE, OC, and UND) only constituted approximately 10%, therefore, we combined these three subtypes when analyzing their associations with IS in order to preserve statistical power. IS-free controls were defined as participants who responded negatively to all eight questions in the Questionnaires for Verifying Stroke-Free Status (QVSFS) and without medical history of IS. The validity and reproducibility of the QVSFS have been reported elsewhere [54,55].

### 4.3. Assessment of Vegetable and Fruit Consumption

Semi-quantitative food frequency questionnaires were used to collect the information of vegetable and fruit consumption by trained interviewers in face-to-face surveys. The sFFQ used in our study was a shortened version of full sFFQ from Shanghai Women’s Health Study (SWHS). The detailed description, validity, and reproducibility of the sFFQs have been reported elsewhere [56]. Briefly, we asked the participants how often, on average, they consumed vegetable and fruit during the previous year (IS-free controls), or the year before onset of ischemic stroke (IS cases). We further asked the participants about their dietary intake levels of vegetables and fruit each time. Dietary intake of vegetables and fruit (grams/day) were estimated by multiplying the reported intake frequency by the dietary intake levels each time.

### 4.4. Genotyping

Fasting venous blood (at least 8 h) was drawn from participants for genotyping. Genomic DNA was extracted from peripheral blood leukocytes with a DNA extraction kit (DP319-01; Tiangen Biotech, Beijing, China). The concentration and purity of DNA were measured by absorbance at 260 nm and 280 nm using UV7501 spectrophotometer. We calculated 260 nm/280 nm ratio (between 1.7 and 2.0) to ensure the DNA quality for subsequent genotyping. The primers were designed according to the sequence of forward strand from dbSNP database and the polymerase chain reaction (PCR) amplifications were performed on the GeneAmp PCR System 9700 instrument (Applied Biosystems, Foster City, CA, USA). *PON1* rs662 was genotyped using time-of-flight mass spectrometry genotyping technology with MassARRAY genetic analysis system (Sequenom Inc., San Diego, CA, USA), and analyzed by MassARRAY Typer Analyzer Version 4.0 (MassARRAY Compact System; Sequenom Inc., San Diego, CA, USA). Blinded duplicates were selected randomly and run along with samples to assess concordance rates of genotyping. The call rate of rs662 in our study was above 95%. We randomly selected one sibling without ischemic stroke from each family to analyze Hardy-Weinberg equilibrium of the SNP using Pearson’s chi-square test and found no violation (*p* > 0.001).

### 4.5. Assessment of Covariates

Demographic and lifestyle characteristics (e.g., age, sex, cigarette smoking, alcohol drinking), medical history (diagnosis of stroke, type 2 diabetes, hypertension, etc.) were collected using structured questionnaires modified from the PhenX Toolkit [57] by trained interviewers in face-to-face surveys. Current smokers were defined as those who smoked at least one cigarette per day and lasting for at least six months. Past smokers were defined as those who regularly smoked in the past but quitted smoking for at least one month. Non-smokers were participants who had never smoked. Current drinkers were defined as those who drank at least 50 milliliters (mL) of liquor per week and lasting for at least six months. Anthropometric variables, such as height and weight, were measured by trained doctors or nurses in a standard procedure. BMI was calculated as weight divided by squared height (kg/m^2^).

### 4.6. Statistical Analysis

Continuous variables, such as age, BMI, vegetable and fruit intake, were described as means and standard deviations. Categorical variables, such as sex, smoking status, alcohol intake, medical history of diabetes and hypertension, were summarized as frequencies. For *PON1* rs662, an additive genetic model was used (0 copy, 1 copy, or 2 copies of G alleles). To minimize the effects of the scale of dietary factors in the interaction analyses, we rescaled vegetable and fruit intake levels by using mean-centered and standardized continuous variables. Conditional logistic regression models, with each family as a stratum, were used to compare the characteristics between IS cases and IS-free controls in order to control for the correlation among family members, and to examine the associations of *PON1* rs662 with IS and its subtypes. Gene-diet interactions were tested by including a cross-product term of dietary intake of vegetable or fruit (mean-centered and standardized continuous variables) by rs662_G allele count in the regression models. We fitted three models when evaluating the interactions: a crude model without any adjustments (Model 1); a multivariate model (Model 2) adjusted for various potential demographic and lifestyle confounding factors, including age (years), sex (male/female), BMI (kg/m^2^), smoking status (never, past, current), alcohol intake (never, past, current), medical history of diabetes (yes/no) and hypertension (yes/no); and a full model (Model 3) further adjusted for vegetable consumption (g/day) when investigating the *PON1*-fruit interaction, and adjusted for fruit consumption (g/day) when investigating the *PON1*-vegetable interaction. We additionally controlled for other dietary factors, such as red meat, egg, milk, and cereal intake in the sensitivity analysis. We visually presented the interactions between *PON1* rs662 and vegetable or fruit intake for both total IS and LAA through figures. All statistical analyses were conducted by STATA (version 13, Stata Corporation, College Station, TX, USA). A two-sided *p* < 0.05 was considered as statistical significance.

## 5. Conclusions

In summary, *PON1* rs662_G was associated with a higher risk of total IS and LAA. Individuals who carry rs662_A allele may benefit to a greater extent from intake of vegetable and thus be more effectively protected from ischemic stroke, whereas carriers of G allele may still remain at greater risk for ischemic stroke due to their genetic backgrounds even when they consume a high level of vegetables. More studies are needed to replicate our findings especially among other populations as paraoxonase activity and allele frequencies vary greatly across diverse populations.

## Figures and Tables

**Table 1:** Characteristics of participants according to ischemic stroke status.

	Ischemic Stroke Cases (*n* = 1007)	IS-Free Controls (*n* = 1151)	*p*-Value
Age (years), mean (SD)	59.3 (8.4)	56.0 (9.2)	<0.001
Male, %	65.3	52.0	<0.001
BMI (kg/m2), mean (SD)	26.3 (3.6)	26.6 (11.2)	0.59
Smoking status, %			0.006
Never smoker, %	40.1	51.8	
Past smoker, %	31.1	22.3	
Current smoker, %	28.8	25.9	
Alcohol drinking, %			0.90
Never drinker, %	55.6	59.2	
Past drinker, %	20.8	15.2	
Current drinker, %	23.6	25.6	
Type 2 diabetes, %	30.0	21.9	<0.001
Hypertension, %	82.7	62.7	<0.001
fruit intake (g/day), mean (SD)	178.9 (209.7)	218.8 (242.7)	0.004
vegetable intake (g/day), mean (SD)	664.7 (941.4)	744.9 (915.2)	0.11
rs662, %			0.008
GG genotype	35.9	39.8	
AG genotype	51.0	48.6	
AA genotype	13.1	11.6	

IS, ischemic stroke; SD, standard deviation, BMI, body mass index.

**Table 2:** Association of *PON1* rs662_G with ischemic stroke and its subtypes.

*PON1* rs662_G	*n*	OR	95% CI	*p*
Ischemic Stroke	1007	1.28	1.07–1.54	0.008
LAA	709	1.32	1.06–1.64	0.013
SAO	185	1.46	0.96–2.22	0.076
Other subtypes ^1^	113	0.80	0.41–1.53	0.495

OR, odds ratio; CI, confidence interval; LAA, large artery atherosclerosis; SAO, small arterial occlusion. ^1^ Other subtypes include cardio embolism (CE), stroke of other determined causes (OC), and stroke of undetermined causes (UND).

**Table 3:** Interaction between *PON1* rs662_G allele numbers and standardized fruit and vegetable intake for total ischemic stroke and LAA.

	Model 1 ^1^			Model 2 ^2^			Model 3 ^3^		
	OR	95% CI	*p*	OR	95% CI	*p*	OR	95% CI	*p*
**Fruit Intake**									
*Ischemic Stroke*									
rs662	1.41	1.02–1.95	0.04	1.39	0.97–2.00	0.07	1.39	0.96–2.01	0.08
Fruit intake	0.66	0.50–0.89	0.006	0.69	0.51–0.94	0.02	0.69	0.50–0.96	0.03
rs662 * Fruit intake	1.13	0.89–1.43	0.31	1.13	0.88–1.46	0.34	1.15	0.89–1.49	0.29
*LAA*									
rs662	1.42	0.99–2.02	0.06	1.33	0.89–1.98	0.17	1.31	0.87–1.98	0.19
Fruit intake	0.66	0.48–0.92	0.01	0.70	0.50–0.99	0.05	0.70	0.48–1.02	0.06
rs662 * Fruit intake	1.08	0.83–1.41	0.58	1.08	0.82–1.44	0.58	1.10	0.82–1.48	0.51
**Vegetable Intake**									
*Ischemic Stroke*									
rs662	1.41	1.13–1.77	0.003	1.39	1.08–1.80	0.01	1.43	0.99–2.07	0.06
Vegetable intake	0.61	0.46–0.81	0.001	0.60	0.43–0.83	0.002	0.73	0.50–1.09	0.12
rs662 * Vegetable intake	1.57	1.19–2.08	0.001	1.59	1.14–2.21	0.006	1.46	0.90–2.35	0.12
*LAA*									
rs662	1.46	1.13–1.88	0.004	1.39	1.04–1.86	0.03	1.33	0.88–2.01	0.17
Vegetable intake	0.64	0.47–0.87	0.004	0.64	0.45–0.91	0.01	0.85	0.57–1.28	0.43
rs662 * Vegetable intake	1.58	1.16–2.14	0.003	1.54	1.08–2.20	0.02	1.13	0.66–1.94	0.65

OR, odds ratio; CI, confidence interval; LAA, large artery atherosclerosis. ^1^ Model 1 without any adjustments. ^2^ Model 2 was adjusted for age (years), sex (male/female), BMI (kg/m^2^), smoking status (never, past, current), alcohol intake (never, past, current), diabetes (yes/no), hypertension (yes/no). ^3^ Model 3 was further adjusted for vegetable consumption (g/day) when investigated the interaction between *PON1* and fruit intake for total ischemic stroke and LAA subtype, and further adjusted for fruit consumption (g/day) when investigated the interaction between *PON1* and vegetable intake for total ischemic stroke and LAA subtype.
